# Determining the Agreement Between Common Measures Related to Vestibulo-ocular Reflex Function After a Mild Traumatic Brain Injury in Children and Adolescents

**DOI:** 10.1016/j.arrct.2022.100217

**Published:** 2022-07-22

**Authors:** Adrienne Crampton, Kathryn J. Schneider, Lisa Grilli, Mathilde Chevignard, Michal Katz-Leurer, Miriam H. Beauchamp, Chantel Debert, Isabelle J. Gagnon

**Affiliations:** a School of Physical and Occupational Therapy, McGill University, Montréal, Quebec, Canada; b Sport Injury Prevention Research Centre, Faculty of Kinesiology, University of Calgary, Alberta, Canada; c Alberta Children's Hospital Research Institute, University of Calgary, Alberta, Canada; d Hotchkiss Brain Institute, University of Calgary, Alberta, Canada; e Montreal Children's Hospital-McGill University Health Centre, Montréal, Quebec, Canada; f Sorbonne Université, CNRS, INSERM, Laboratoire d'Imagerie Biomédicale, Paris, France; g Sorbonne Université, Handicap Moteur et Cognitif et Réadaptation, Paris, France; h Rehabilitation Department for Children with Acquired Neurological Injury and Outreach Team for Children and Adolescents With Acquired Brain Injury, Saint Maurice Hospitals, Saint Maurice, France; i Physical Therapy Department, University of Tel-Aviv, Tel-Aviv, Israel; j Ste-Justine Hospital Research Center, Montréal, Quebec, Canada; k Department of Psychology, University of Montréal, Montréal, Quebec, Canada; l Department of Clinical Neuroscience, University of Calgary, Alberta, Canada

**Keywords:** Brain concussion, Brain injuries, traumatic, Reflex, vestibulo-ocular, Rehabilitation, Vision, ocular, cDVA, computerized dynamic visual acuity, CFRT, cervical flexion-rotation test, HTT, head thrust test, mTBI, mild traumatic brain injury, PCSI, Postconcussion Symptom Inventory, PedsQL, Pediatric Quality of Life Inventory, ROM, range of motion, TBI, traumatic brain injury, vHIT, video head impulse test, VOMS, Vestibular/Ocular-Motor Screening, VOR, vestibulo-ocular reflex

## Abstract

**Objective:**

To (1) determine the level of agreement between symptom provocation and performance-based tests of vestibulo-ocular reflex (VOR) function after pediatric mild traumatic brain injury (mTBI) and (2) describe the level of symptom provocation induced by a VOR task in individuals with and without cervical findings.

**Design:**

Cross-sectional.

**Setting:**

This study was conducted at a tertiary care pediatric hospital.

**Participants:**

A total of 101 participants (N=101) aged 6-18 years within 3 weeks of mTBI diagnosis were included (54.5% female; mean age, 13.92±2.63 years; mean time since injury at assessment, 18.26±6.16 days).

**Interventions:**

None.

**Main Outcome Measures:**

*Symptom provocation* (Vestibular/Ocular Motor Screening tool), *performance* (clinician-observed VOR performance, head thrust test [HTT], computerized dynamic visual acuity test, video head impulse test), and c*ervical impairment* (cervical flexion-rotation test, range of motion test, self-reported neck pain). Agreement was evaluated using Cohen's κ statistic.

**Results:**

No outcomes demonstrated agreement with symptom provocation (κ=−0.15 to 0.14). Fair agreement demonstrated between clinician-observed VOR performance and HTT (κ=0.32), with little to no agreement demonstrated between other measures. Proportions reporting test-induced dizziness and headache were greater among individuals with cervical findings (29.1%-41.8%) than without (2.3%-6.8%).

**Conclusions:**

Findings support that symptom provocation and performance-based tests measure different constructs and thus have distinct roles when assessing VOR function. Findings suggest results from measures of symptom provocation may be influenced by coexisting cervical impairments, underlining the value of assessing for cervical injury after pediatric mTBI.

## Highlights

• A battery of complementary tests is needed to assess vestibulo-ocular reflex (VOR) in pediatric mild traumatic brain injury (mTBI).
• This battery should include both symptom- and performance-based measures.
• Best practice recommendation is needed for such a battery in clinical settings.
• Cervical injury presence may influence symptoms induced during VOR testing.
• There is value of assessing for cervical injury post pediatric mTBI.

Pediatric traumatic brain injury (TBI) incidence across the globe has been estimated between 47 and 280 per 100,000 children, with more than 80% being mild traumatic brain injuries (mTBIs).^1^ While symptoms after mTBI are unique to each child and adolescent, difficulties specific to vestibulo-ocular reflex (VOR) function have been identified in 29%-69% of children and adolescents.^2^, ^3^, ^4^, ^5^ These difficulties can interfere with academic, sport, and recreation activities and may contribute to poor prognosis after mTBI because VOR integrity is essential to maintaining gaze stability during head motion. It does so by facilitating eye movements in equal and opposite direction to head movements. The primary sensory input acting to trigger the VOR motor response originates from the semicircular canals, situated in the peripheral sensory apparatus within the inner ear, which detects angular acceleration in the 3 planes of motion.^6^, ^7^, ^8^ The semicircular canals are in turn interconnected with the visual system as well as with both central and peripheral structures involved in processing and coordinating the VOR response.

Identifying how to best evaluate for the presence of VOR dysfunction in children and adolescents and understanding the pathophysiology underlying the reported difficulties is important for clinicians and researchers seeking to develop relevant treatment strategies. To date, a variety of different measures have been developed and are used to assess the VOR.

In specialized clinics, criterion standard vestibular tests such as rotary chair and caloric testing^9^ are often used; however, these are not able to evaluate the VOR at high frequencies and can be poorly tolerated by symptomatic patients. In more general clinical contexts, there is little consensus regarding best practice when assessing VOR function in populations with mTBI with tests measuring different elements of VOR function as well as varying in their cost, ease of use, and accessibility.^10^ Common clinical tests often require little equipment, are low cost, and are accessible to both practitioners and patients; however, they present shortcomings regarding their precision and ability to quantify deficits observed.^11^ Conversely, computerized versions of tests are more precise but may not be accessible and/or available because they are more expensive, can be cumbersome, and require training to administer and expertise to interpret. Finally, measures of symptom provocation in response to VOR testing are low in cost and easy to administer; however, in the past these focused more on the symptoms induced by a VOR test than on VOR function itself. Performance on these measures may also be influenced by the presence of coexisting injuries such as cervical impairments,^12^, ^13^, ^14^ which can also be associated with dizziness and other visuovestibular symptoms after mTBI.^13^, ^14^, ^15^ Because measures of symptom provocation in response to VOR tasks have more recently started to be used to identify the presence of potential vestibulo-ocular impairments,^16^, ^17^, ^18^ these influences need to be better understood.

Despite previous studies focusing on the psychometric properties of clinical and computerized performance-based VOR tests as well as measures of symptom provocation in response to VOR testing in pediatric populations,^19^, ^20^, ^21^, ^22^ to our knowledge none report on agreement between commonly used measures of VOR function in pediatric mTBI. Determining agreement can contribute to both clinical practice and scientific knowledge because it may inform a more strategic selection of complementary tests, supporting a comprehensive assessment of VOR function. This would be beneficial from a time, tolerability, energy, and economic perspective. The focus of this study will be specifically on mTBI because it constitutes the largest portion of TBIs reported among children and adolescents. The first objective of this study was therefore to determine the level of agreement between measures of symptom provocation in response to VOR testing and performance-based tests of VOR function in a pediatric population with mTBI. We hypothesized that of the outcome measures included, only clinical and computerized measures assessing similar elements of VOR function would demonstrate agreement, while remaining measures would not. To explore the potential influence of cervical impairments on the presence of symptoms during VOR tasks, the second objective was to describe the level of symptom provocation induced by a VOR task in individuals with cervical findings and those without. We hypothesized that individuals with cervical findings would report higher levels of symptom provocation.

## Methods

This cross-sectional study included a consecutive convenience sample of participants recruited prospectively from the emergency department at a tertiary care pediatric hospital, the Montréal Children's Hospital (McGill University Health Center) in the emergency department and at the Institution's Concussion Clinic, the University of Calgary Sport Medicine Centre, and the Acute Sport Concussion Clinic (University of Calgary). The study was approved by the pediatric panel of the pediatric panel of the McGill University Health Center Research Ethics Board and by the Conjoint Health Research Ethics Board at the University of Calgary. All participants provided written informed consent (parent) and assent (child) to the study.

### Participants

This study was a substudy of a larger multicenter international project (the SiMPLy Rehab initiative). Participants enrolled in the larger project, aged 6-18 years and assessed within 3 weeks of sustaining a physician-diagnosed mTBI (as defined in the American Congress of Rehabilitation Medicine and the World Health Organization collaborating task force on mTBI),^23^ were included in this study (up to 7 days beyond the 3-week limit was permitted if necessary, participants remained in the subacute stage of recovery). Diagnosis of mTBI (as referenced) was aligned with current best practice in the Québec trauma system. Participants were excluded if 1 or more of the following was present: (1) history of TBI in the preceding 6 months or any previous TBI with unresolved symptoms and/or impairments; (2) presence of comorbidities that would restrict, negatively influence, or prevent the participant's ability to complete the study protocol (ie, spinal cord injury; orthopedic or neurologic condition; severe visual, vestibular, or auditory deficit); (3) use of medications that affect the vestibular system; or (4) consent to participate in the study but withdrawal prior to assessment. All participants received standard acute care by family physicians, pediatricians, walk-in clinics, or the emergency department. Because this substudy was part of the previously mentioned larger parent study, sample size calculations were performed for the latter.

### Procedures

Assessments took take place at the mTBI/Concussion program within the Montreal Children's Hospital or at the Concussion Lab at the University of Calgary between December 2017 and June 2020. Medical history of participants was obtained through a medical chart review form upon enrollment. Prior to arrival for their assessment, participants were asked to complete developmentally appropriate versions of patient-reported outcome measures (Pediatric Quality of Life Inventory [PedsQL]^24^ and Postconcussion Symptom Inventory [PCSI]^25^) to thoroughly characterize the sample. An evaluator (trained by a certified physiotherapist) performed the battery of assessments at each site, including symptom-based and performance-based measures of VOR function as well as measures assessing cervical spine function. Training for evaluators was developed by expert clinicians and researchers, which took place prior to the start of the study, while ongoing consultation was available as needed. This included a standardized testing manual, videos of measures included, in-person practice with trained physiotherapists, and continual discussion among evaluators to ensure homogeneity across sites throughout the study.

### Outcome measures

Five outcome measures were administered from 4 VOR tests focusing on different commonly assessed elements of, or relating to, VOR function. Gaze stability in response to voluntary head movements was assessed clinically by looking at alterations in the child's or adolescent's performance on the VOR task included in the Vestibular/Ocular-Motor Screening (VOMS) tool^22^^,^^26^ and with a computerized dynamic visual acuity (cDVA) test.^27^ Symptom provocation in response to VOR tasks requiring eye head movements was assessed using the same VOMS tasks. Gaze stability in response to unplanned passive high velocity head movements was assessed with the clinical head thrust test (HTT)^28^^,^^29^ and with a similar computerized test, the video head impulse test (vHIT).^30^ See table 1 for full description of outcome measures.

**Table 1:** Description of outcome measures included in agreement analysis

VOR Outcome Measure	Procedure and Justification for Test	Definition
VOMS^22^^,^^24^ VOR items Symptom provocation	The VOMS was developed as a standardized screening tool to assess symptom provocation (headache, dizziness, nausea, fogginess) in response to common VOR and outcome measure tasks in individuals after concussion. It includes 7 tasks: smooth pursuits, vertical saccades, horizontal saccades, convergence, vertical VOR, horizontal VOR, and visual motion sensitivity. For the purpose of this study only the vertical and horizontal VOR tasks were included. As per test instructions, the participants were asked to face the examiner and rotate their head 20 degrees to each side at a rate of 180 bpm first horizontally while maintaining a focus on the examiner's nose. Ten repetitions (back and forth) were performed. This was repeated in the vertical direction.	Symptom provocation was considered to be present or abnormal when the participant reported experiencing an increase ≥2 points on any of the 4 symptoms rated for either or both the horizontal or vertical VOR tasks.
Clinician-observed VOR performance on VOMS VOR items	As above. For the purpose of this study, a quantified component was added. The evaluator observed the performance of participants during the task described above and noted the presence of corrective saccades (yes/no) during either or both horizontal and vertical VOR tasks.	Presence of saccade was considered abnormal VOR performance.
HTT^27^	The HTT often used as part of clinical examinations to identify individuals with peripheral vestibular hypofunction^27^ was included as a clinical measure of VOR function in the context of unplanned high-velocity head movements. In this test, the evaluator administered quick, small amplitude and unpredictable high-acceleration head rotations.^28^ The participants were instructed to maintain their gaze on the evaluator's nose.	Presence of catch-up saccades as observed by the assessor indicated abnormal VOR function.
vHIT, ICS software^29^	The vHIT was performed using the ICS Impulse software (Natus) to assess the horizontal semicircular canals and as a computerized alternative to the HTT. The participants sat facing the wall and maintained their gaze on a fixation dot, while the tester rotated the participants’ head horizontally 10-20 degrees in a short abrupt manner, unpredictably to the left and right.^29^	The mean gain of the VOR was used for analysis with an abnormal cutoff of <0.8.
cDVA, InVision system	The cDVA test was performed using the NeuroCom InVision System (Natus); cDVA testing was selected because it has demonstrated high positive predictive value (96%) for individuals with vestibular disorders (unilateral vestibular loss and bilateral vestibular hypofunction) and high negative predictive values (93%) for those without.^26^ One limitation is that psychometric properties for this test remain to be determined across younger pediatric populations. The participants first completed the static visual acuity test through a series of tumbling E displays of varying sizes determined by an algorithm while sitting 10 feet from the screen. A head tracking device to capture head velocity was then placed on the participants’ head and the DVA test was performed with fixed minimal velocity active head rotations at 120 deg/s.	Abnormal DVA change when comparing static visual acuity and DVA was considered >0.3 logarithm of the minimal angle of resolution.

Five outcome measures from 3 tests were also included to assess cervical spine function, measuring motion limitation as well as self-reported pain. These included the cervical flexion-rotation test (CFRT), the cervical range of motion (ROM) test, and self-reported neck pain.

The CFRT^31^^,^^32^ assessed performance (abnormal if range of motion was limited or firm resistance encountered, evaluator interpretation) and any presence of pain (abnormal if pain was present, patient self-report).

The cervical ROM test assessed performance (abnormal if limited ROM was present on active rotation, side flexion, flexion or extension, evaluator interpretation) and any presence of pain (abnormal if pain was present, patient self-report).

A numeric pain scale assessed participants’ self-reported neck pain within the past 48 hours. A scale from 0-2 was used for children younger than 13 years and from 0-6 for adolescents 13-18 years old (abnormal if patient reported any neck pain that was not present prior to mTBI. Data were dichotomized to fulfill our second more descriptive objective.

### Analysis

Given the binary nature of the data, agreement among symptom-based and performance-based measures was assessed using Cohen's κ coefficients (interpreted such that ≤0 indicates no agreement, 0.01-0.20 none to slight, 0.21-0.40 fair, 0.41-0.6 moderate, 0.61-0.80 substantial, 0.81-1.00 almost perfect) to account for the possibility of agreement occurring by chance and thus provide more robust measures than a simple percentage agreement calculation. To address our second objective, participants were categorized into 2 subgroups according to results from the 5 outcome measures assessing cervical spine function. The presence of pain or abnormal function on 1 or more cervical spine measures categorized the participant into the Cervical group (with cervical findings). The absence of clinical findings on any of these measures categorized the participant into the None group (without cervical findings). Descriptive data were used rather than a formal statistical test to address objective 2 because it was exploratory in nature.

## Results

Our sample consisted of 101 participants (54.5% female) (table 2), with a mean ± SD age of 13.92±2.63 years (range, 7-17 years) and mean time from injury to VOR assessment of 18.26±6.61 days (range, 2-38). Concerning injury mechanism, 70.3% of participants in our sample sustained their mTBI from sport and 29.7% from recreational play or other reasons. Regarding prior mTBI history, 58.0% of our sample did not have previous history of injury. Mean scores on patient-reported outcome measures were low indicating possible natural recovery after injury (PCSI ranged from 1.5/10±1.92 to 26.38/120±24.20). Scores on the PedsQL ranged from 92.39±4.16 to 71.92±17.28 of 100). Pain was reported during assessment of cervical ROM in 26.0% of our sample, during the CFRT (13.1% right and 15.2% left), and through self-report on the numeric pain scale (45.7%). Because the PCSI^25^ and PedsQL^24^ provide separate versions for ages 5-7, 8-12, and 13-18 years, total scores were presented according to age group (see table 2). Seven participants were assessed beyond 28 days because of a protocol deviation. Raw data were verified for these individuals and they did not present as outliers. Additionally, elevated perception times were noted in 5 individuals during the cDVA assessment, which may have affected the validity of their DVA logarithm of the minimal angle of resolution values.

**Table 2:** Descriptive characteristics and additional outcome measures

Outcome	Mean ± SD or %	N*
Descriptive characteristics		
Age (y), mean ± SD	13.92±2.63	101
Sex, male (%)	45.5	101
Time from mTBI to assessment (d), mean ± SD	18.26±6.16	101
Any psychiatric disorder (%)†	10.0	101
Any developmental disability (%)‡	14	101
Previous history of concussion (%)	42.0	101
mTBI from a sport (%)	70.3	99
mTBI from recreation, other or unspecified (%)	29.7	99
Mechanism of injury (%)		99
Sports	70.3	
Recreational play or other	27.2	
Unknown	2.0	
Postconcussion symptoms		
PCSI total score, mean ± SD§		95
5-7 y (max score 10)	1.5±1.92	
8-12 y (max score 34)	6.95±8.24	
13-18 y (max score 120)	26.38±24.20	
Dizziness present on PCSI (%)	51.1	94
Cervical examination		
Normal cervical ROM (%)	94.0	100
Pain present on cervical ROM (%)	26.0	100
Normal cervical flexion-rotation right (%)	94.9	99
Normal cervical flexion-rotation left (%)	94.9	99
Cervical flexion-rotation pain right (%)	13.1	99
Cervical flexion-rotation pain left (%)	15.2	99
Self-reported neck pain present post injury (%)	45.74	94
≥1 of above neck observations present (%)	55.44	101
Global outcome		
PedsQL total score, mean ± SD‖		90
5-7 y (max score 100)	92.39±4.16	
8-12 y (max score 100)	81.06±15.98	
13-18 y (max score 100)	71.92±17.28	

⁎ Total no. of participants who completed the assessment.

† Defined as any of the following: anxiety, depression, sleep disorder or other.

‡ Defined as any of the following: learning disability, attention-deficit hyperactivity disorder, developmental disorder.

§ Postconcussion symptom inventory.

‖ Pediatric quality of life questionnaire.

### Performance on outcome measures assessing VOR function

On performance-based measures, few participants had clinician-observed corrective saccades during the assessment of VOR performance on VOMS VOR items (1/98) and the HTT (5/98), and few demonstrated abnormal VOR gain ratios on the vHIT (right/left average 3/88). Conversely, many participants demonstrated abnormal performance on the cDVA test (31/80). When considering measured symptom provocation induced on the VOMS VOR test, 29/93 participants reported a symptom increase of ≥2 on 1 or both tasks. Performance N values represent the number of participants who completed each individual assessment.

### Level of agreement between measures of symptom provocation in response to VOR testing and performance-based measures

No performance-based outcomes demonstrated more than slight agreement with symptom provocation in response to VOR testing (κ range, −0.15 to 0.14) (table 3).

**Table 3:** Agreement between symptom provocation in response to VOR testing and measures of VOR function

Measure	Cohen's κ With Symptom Provocation in Response to VOR Testing	95% CI
Clinician-observed VOR performance on VOMS VOR items	0.05	−0.04 to 0.14
Head thrust test	0.11	−0.03 to 0.26
ICS Impulse left vHIT gain	−0.15	−0.25 to −0.06
ICS Impulse right vHIT gain	−0.05	−0.11 to 0.02
ICS Impulse average vHIT gain	−0.07	−0.14 to <0.01
InVision left DVA	−0.04	−0.25 to 0.18
InVision right DVA	0.07	−0.15 to 0.29
InVision average vHIT	0.14	−0.09 to 0.36

### Level of agreement between performance-based measures

Fair agreement was observed between clinician-observed VOR performance on VOMS VOR items and the clinical HTT (κ=0.32). All other comparisons showed poor to slight (defined as up to 0.2) agreement (table 4).

**Table 4:** Kappa values between performance-based outcomes

Outcome	Performance on VOMS VOR items (95% CI)	HTT(95% CI)	vHIT(95% CI)	cDVA(95% CI)
Clinician-observed performance on VOMS VOR items		0.32 (−0.16 to 0.80)	NA	NA
HTT	0.32 (−0.16 to 0.80)		−0.04 (−0.07 to −0.01)	0.05 (−0.06 to 0.17)
vHIT	NA	−0.04 (−0.07 to −0.01)		0.08 (−0.03 to 0.18)
cDVA	NA	0.05 (−0.06 to 0.17)	0.08 (−0.03 to 0.18)	

Abbreviations: cDVA, computerized dynamic visual acuity; HTT, head thrust test; NA, not applicable; vHIT, video head impulse test.

### Symptom provocation according to cervical findings

**Fig 1:**
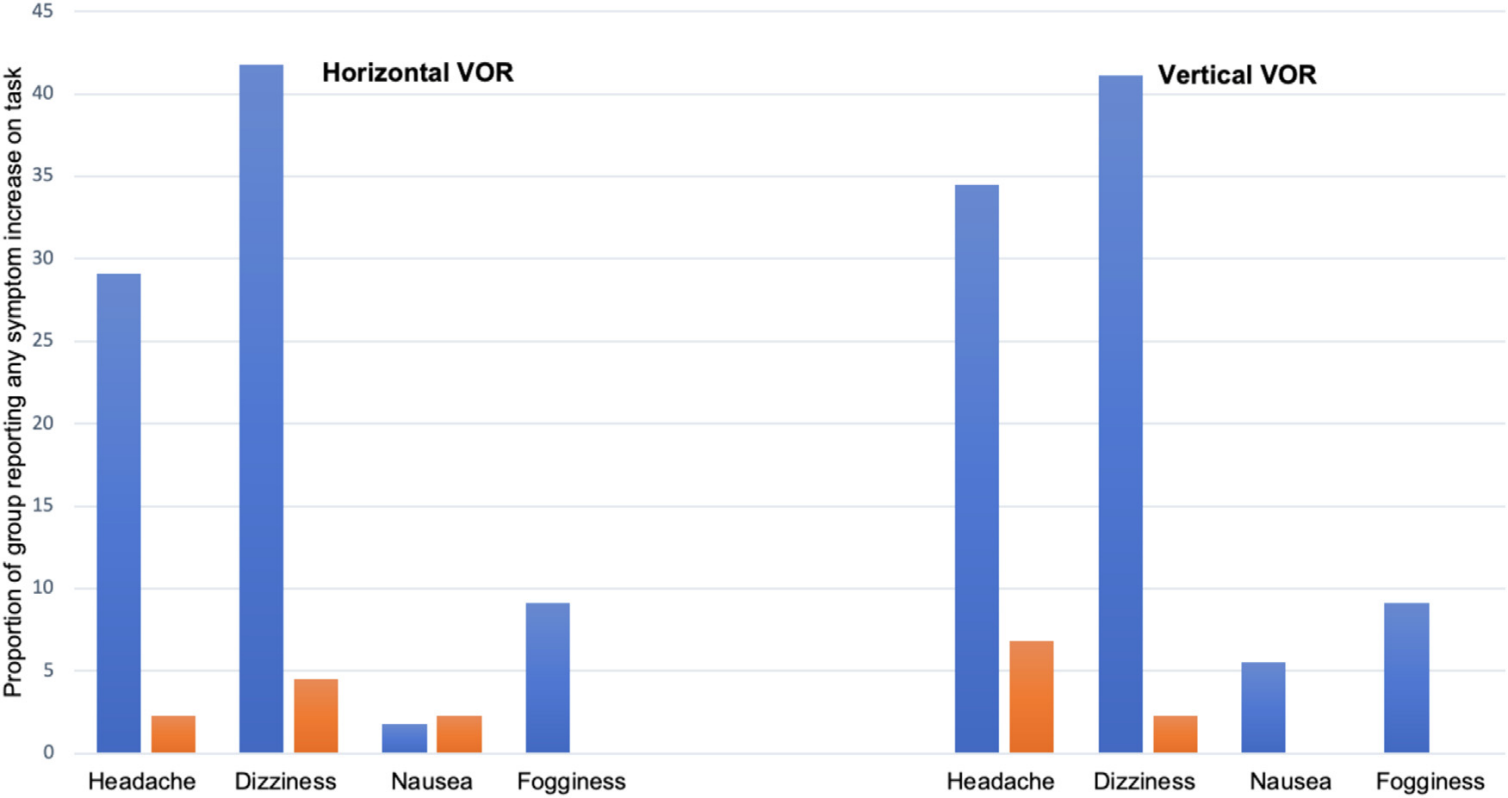
Proportion of sample reporting symptoms at rest and provocation with testing by subgroup (Cervical* vs None).

NOTE. Blue=Cervical (n=56); orange=None (n=44). *CERVICAL sample, n=55 for horizontal and vertical symptom provocation and n=54 for horizontal VOR nausea.

To address the second study objective, the sample was separated into 2 groups: Cervical and None (previously described). Headache and dizziness were the most frequent symptoms provoked during both the horizontal and vertical VOMS VOR items (table 5), and a higher proportion of participants in the Cervical group reported such symptoms (fig 1).

**Table 5:** Symptom provocation in response to VOR* testing by symptom type

Variable	Headache	Dizziness	Nausea	Fogginess	Total change
Horizontal VOR (mean symptom change*)	0.49	1.21	0.02	0	1.70
Horizontal VOR (total symptom change†)	24	60	1	0	84
n	98	98	97	98	98
Vertical VOR (mean symptom change*)	0.75	1.07	0.02	0.12	1.94
Vertical VOR (total symptom change†)	37	53	1	6	96
n	98	98	97	98	98

⁎ Mean symptom change: sample mean symptom increase reported by symptom type after VOR task.

† Total symptom change: sum of symptom increase reported by all participants after VOR task.

## Discussion

The first objective was to determine the level of agreement between measures of symptom provocation in response to VOR testing and performance-based tests of VOR function in a pediatric population with mTBI. The second objective was to characterize the level of symptoms provoked by VOR tests in individuals with cervical findings and those without. Considering (1) approximately one-third of participants reported significant symptom provocation in the context of the VOMS VOR test; (2) there was lack of agreement between measures of symptom provocation and performance-based tests; and (3) the subgroup symptom differences suggested potential contributions from coexisting cervical impairments, it appears that symptom provocation induced by VOR testing may not measure the same construct as performance-based outcome measures of VOR function.

### Comparing performance-based measures

Across performance-based measures included, fair agreement was demonstrated between clinician-observed VOR performance on VOMS VOR items outcome and the clinical HTT (κ=0.32). This may indicate that despite the contributions from additional systems and central preprogramming during active voluntary head movements, positive performance findings on the VOR test portion of the VOMS may still be sensitive and specific to the VOR because the HTT uses unplanned, passive head movements that better isolate VOR function. These results should be further explored.

### Comparing clinical and computerized measures

In this study, the agreement between a clinical (HTT) and a computerized (vHIT) measure of gaze stability during unplanned, passive high-velocity head movements was assessed, and no agreement was observed (κ=−0.04). Given the vHIT is increasingly used in populations with mTBI and TBI,^33^^,^^34^ this finding can contribute to discussions surrounding the added value of the vHIT to clinical practice. Perhaps, if agreement is lacking, the value may not lie in substituting the vHIT for the HTT but rather in using them for different purposes. The HTT could be used to obtain a more general, functional measure of the VOR, while the vHIT could provide information on multiple variables, ultimately allowing characterization of the VOR response with a higher level of detail.

### Symptom provocation and cervical spine findings

Participants demonstrated pain on cervical flexion in 13.1% and 15.2% (right, left) of cases on the cervical ROM test in 26.0%, and 45.7% self-reported neck pain. To further understand the symptom provocation induced by VOMS VOR testing, symptom provocation was described by subcomponent. The data suggest that headaches and dizziness are predominant. When further characterizing symptom provocation induced by VOMS VOR tests by group (Cervical vs None), a noticeable difference seemed present with a larger proportion in those with cervical findings who reported headache and dizziness symptoms induced by VOMS VOR testing. It is possible that certain symptoms may be a result of physiological impairments to the cervical spine and/or associated pathways rather than alterations to the VOR. The forceful mechanisms leading to mTBI can often simultaneously affect the highly mobile cervical region of the spine and compromise afferent input in the cervical region, which can contribute to various symptoms, including dizziness and visual impairments.^35^ The findings align with those of previous studies that have identified cervical impairments as a potential contributor to symptoms such as headaches and dizziness.^12^^,^^13^^,^^36^ Considering the cervical spine region when assessing individuals and developing targeted treatment plans could result in more favorable prognosis and overall recovery.^12^^,^^13^^,^^36^

### Study limitations

A general limitation of this study is the sampling method used. This method could have induced some bias as individuals recruited in the specialized clinical settings outlined (emergency department, concussion clinics, and a sports medicine center) may be experiencing more complicated recoveries. However, this was addressed in our analysis and by the multicentered nature of the study, which expanded the type of participant included. Nevertheless, the generalizability of our findings should be restricted to similar patient populations.

A more specific limitation of this study is that because limited abnormal findings were identified in certain outcome measures, κ values informing the agreement reported among these measures must be considered cautiously. Future studies with larger sample sizes should further explore and confirm findings. Because scores on both the PedsQL and PCSI were relatively low, some natural recovery in this patient sample may have occurred and contributed to the limited abnormal observations on outcome measures. A shorter delay between assessment and injury would be favorable in future research.

Regarding individual measures, the high proportion of participants demonstrating abnormal results on the cDVA test is unexpected. Because opinions on the reliability of the InVision DVA test are not uniform,^20^^,^^37^^,^^38^ these findings should be interpreted with caution and support the need to further explore the psychometric properties of the InVision DVA test when used in a pediatric population with mTBI and across all ages. Finally, consensus regarding the optimal amount of head impulses required when administering the vHIT in pediatric populations has not yet been reached. As such, future studies should refer to the most recent literature and recommendations when administering this measure.

## Conclusions

This study failed to show agreement between measures of symptom provocation in response to VOR testing and performance-based measures of VOR function and suggests there is value in including both types of measures when assessing VOR function in pediatric populations with mTBI. Findings suggest results from such measures of symptom provocation may be influenced by coexisting cervical impairments. Acquiring a more precise understanding of this relationship would be beneficial because symptom-based measures could prove useful in flagging additional systems that may influence certain disabling sensations reported (ie, dizziness, headaches). Additional research specifically outlining how each included measure can contribute to the comprehensive assessment of VOR function in pediatric mTBI would benefit clinicians in selecting appropriate tests, interpreting these, and in turn planning appropriate interventions.
